# Research progress and implications of the application of large language model in shared decision-making in China’s healthcare field

**DOI:** 10.3389/fpubh.2025.1605212

**Published:** 2025-07-10

**Authors:** Xuejing Li, Sihan Chen, Meiqi Meng, Ziyan Wang, Hongzhan Jiang, Yufang Hao

**Affiliations:** ^1^School of Nursing, Beijing University of Chinese Medicine, Beijing, China; ^2^Evidence-Based Nursing Research Center, School of Nursing, Beijing University of Chinese Medicine, Beijing, China; ^3^Best Practice Guidelines Research Center, Registered Nurses' Association of Ontario, BPSO, Toronto, ON, Canada

**Keywords:** large language models, shared decision making, decision support, knowledge localization, review

## Abstract

Shared Decision Making (SDM), as a modern medical decision-making model emphasizing patient participation, faces multidimensional challenges in China, including uneven distribution of medical resources, knowledge gaps, and inadequate cultural adaptation. The implementation of SDM in China is hindered by time constraints, insufficient patient willingness to participate, a lack of standardized decision support tools, and structural barriers such as healthcare reimbursement systems. Large Language Models (LLMs), with their powerful natural language processing capabilities, demonstrate unique advantages in enhancing communication efficiency, supporting personalized decision-making, and promoting multi-party collaboration. Key functionalities such as information integration, personalized support tools, and sentiment analysis significantly improve patient engagement and decision quality. However, LLMs still face limitations in localization, decision-chain completeness, and handling complex scenarios, particularly in understanding traditional Chinese medicine (TCM) knowledge and supporting family-oriented decision-making models. Future efforts should focus on constructing integrated knowledge graphs of biomedicine and Traditional Chinese Medicine, optimizing multi-layered expression capabilities, and improving model interpretability to promote LLMs’ in-depth application in SDM within China, ultimately enhancing healthcare quality and patient satisfaction.

## 1 Introduction

Shared Decision-Making (SDM) is a modern healthcare decision model that emphasizes collaboration between healthcare providers and patients, playing a crucial role in improving healthcare service quality and enhancing physician-patient relationships (1). SDM is the process in which healthcare providers, while presenting different clinical options and detailed information on their outcomes based on medical evidence, incorporate the patient’s values and decision-making preferences, encouraging the patient’s involvement in the medical decision-making process and actively listening to their perspective (2). However, studies indicate that more than 31.6% of healthcare providers have yet to engage in the SDM processes in clinical practice (3). The main barriers include a significant knowledge gap between physicians and patients, traditional physician-patient communication paradigms that lead most patients to passively accept medical advice rather than actively participate in decision making, and the lack of standardized decision support tools (4, 5). Additionally, systemic factors such as strained medical resources and healthcare reimbursement mechanisms further constrain healthcare providers’ enthusiasm for implementing SDM (6, 7).

In recent years, the rapid development of artificial intelligence (AI) technologies, particularly Large Language Models (LLMs), has introduced new possibilities for addressing these challenges. LLMs are artificial intelligence models specifically engineered for comprehending and generating natural language. Typically built on deep learning architectures like Transformer (8) and trained on massive text data. They exhibit remarkable language processing capabilities, representing a specific category of AI technology dedicated to natural language processing (NLP).

LLMs equipped with NLP technology can provide an informational foundation for SDM by virtue of their powerful text extraction and integration capabilities. Beata F. et al. developed the ExECT program using NLP to extract key epilepsy-related information—including diagnosis, seizure type, and seizure frequency—from 200 outpatient clinic letters. The results demonstrated high performance metrics for each information category, with overall precision, recall, and F1 score of 91, 81%, and 86%, respectively (9), validating the superior information processing capabilities of NLP. One of the fundamental characteristic of SDM is the bidirectional sharing of information (10). Due to the knowledge gap, professional medical knowledge may be incomprehensible from the patient’s perspective. LLMs can bridge this gap by providing accurate and easy-to-understand information, thereby holding great potential in strengthening healthcare communication (11). Research evaluating the ability of LLMs to generate patient education materials found that Google Gemini achieved a readability score of 68.7, indicating that LLM-generated educational materials are readable and understandable for most patients (12).

Foreign scholars have focused on the application of AI in decision-making since 2017 (13). Giorgino et al. explored the potential of ChatGPT in orthopedic practice, finding that ChatGPT could facilitate communication with orthopedic patients, assist in clinical decision-making, and improve patient communication and education (14). Lawson McLean et al. assessed the potential of LLMs in SDM from a neuro-oncology perspective, suggesting that the appropriate integration of LLMs could enhance patient engagement and improve the physician-patient relationship (15). These researches have primarily focused on the effectiveness of LLMs in decision-making, summarizing the possibilities of these technologies in SDM. The MERGE (Merge Evidence-based Research and artificial intelliGence to support smart dEcision) working group in China reviewed the current application status from the perspectives of AI development, testing, and implementation in the SDM field, discussing the potential challenges of AI-assisted decision support (16). However, this study focuses more on discussions at the theoretical level of the entire AI technology, and lacks analysis targeting the practical application directions and future development of LLMs. Based on the above analysis, although existing research has revealed the value of LLMs in clinical decision-making and some SDM scenarios, there is a lack of in-depth discussion regarding their practical applications, adaptability within the Chinese healthcare system, and future development directions, particularly the application research within China’s healthcare system remains scarce.

Therefore, this study focuses on the application of LLMs in SDM practices, reviewing the current implementation status, identifying challenges and suggesting improvements within the context of Chinese healthcare system, with the aim of providing theoretical guidance and practical support for the scientific advancement of SDM in China.

## 2 Methodology

This study employed a narrative review methodology with a multi-database search strategy covering major academic databases in both Chinese and English. The Chinese databases included China National Knowledge Infrastructure (CNKI) and Wanfang Database, while the English databases comprised PubMed, Web of Science Core Collection, and Scopus. The search timeframe was established from January 1, 2014, to December 31, 2024.

The Chinese search strategy utilized the following combination: (“大型语言模型” OR “大语言模型” OR “LLM” OR “ChatGPT” OR “GPT” OR “人工智能”) AND (“共同决策” OR “医患共同决策” OR “共享决策” OR “医疗决策” OR “临床决策”). The English search strategy was: (“Large Language Model*” OR “LLM*” OR “ChatGPT” OR “GPT-4” OR “Generative AI” OR “Natural Language Processing”) AND (“Shared Decision Making” OR “SDM” OR “Clinical Decision Making” OR “Medical Decision Making” OR “Patient Decision Support”). The search strategy underwent preliminary optimization through pilot searches and was validated by library information specialists prior to formal implementation.

The inclusion criteria encompassed: (1) Study types: peer-reviewed original research, systematic reviews, meta-analyses, case studies, and technical reports; (2) Research topics: studies explicitly involving the application of large language models in medical decision support, including but not limited to information extraction, decision support tool development, and patient education functions. The exclusion criteria were established as: (1) Publication types: conference abstracts, brief reports, editorials, correspondence articles, and preprints; (2) Research quality: non-peer-reviewed literature or studies with apparent methodological deficiencies; (3) Accessibility: literature for which full text could not be obtained.

Literature screening employed a two-stage independent screening method. In the first stage, two researchers independently conducted title and abstract screening using standardized screening forms to document the decision-making process. The second stage involved full-text evaluation of potentially relevant literature, with strict application of inclusion and exclusion criteria. Disagreements during the screening process were resolved through discussion, with consultation of third-party expert opinion when necessary.

## 3 Result

### 3.1 Literature selection process

**Figure 1:**
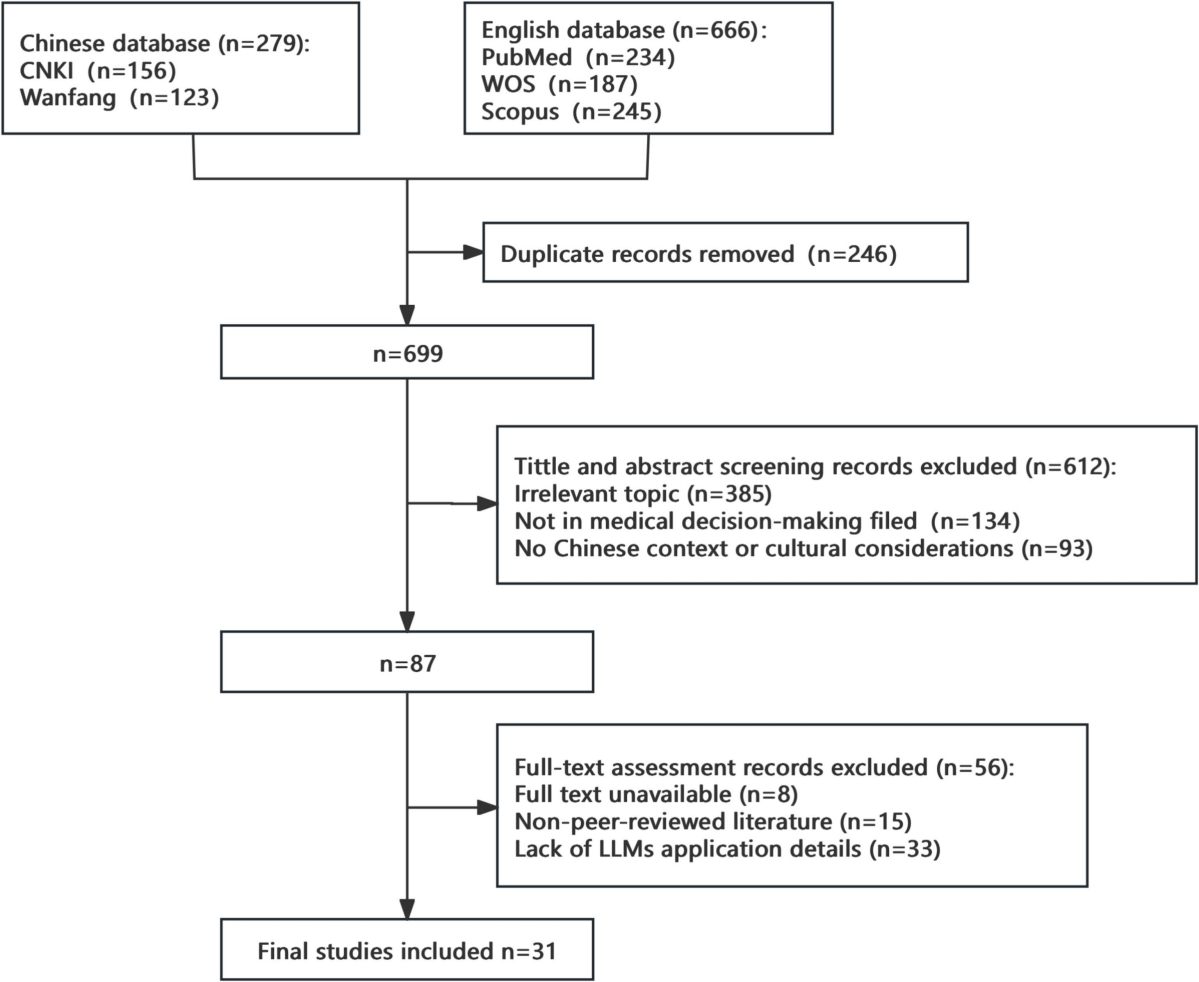
Literature selection process.

Study selection process is detailed in Figure 1.

### 3.2 Barriers to the implementation of SDM in China

Robust SDM theoretical frameworks and evidence-based Decision Aids (DAs), supported by mature clinical protocols, policy infrastructure, and multisectoral support systems in foreign countries, establish a substantive foundation for leveraging LLMs in SDM applications. Conversely, SDM research in China remains at the exploratory stage, with implementation confronting multidimensional systemic challenges. Identifying these barriers will enable critical assessment of LLMs’ capacity to bridge context-specific SDM implementation gaps in China.

**Figure 2:**
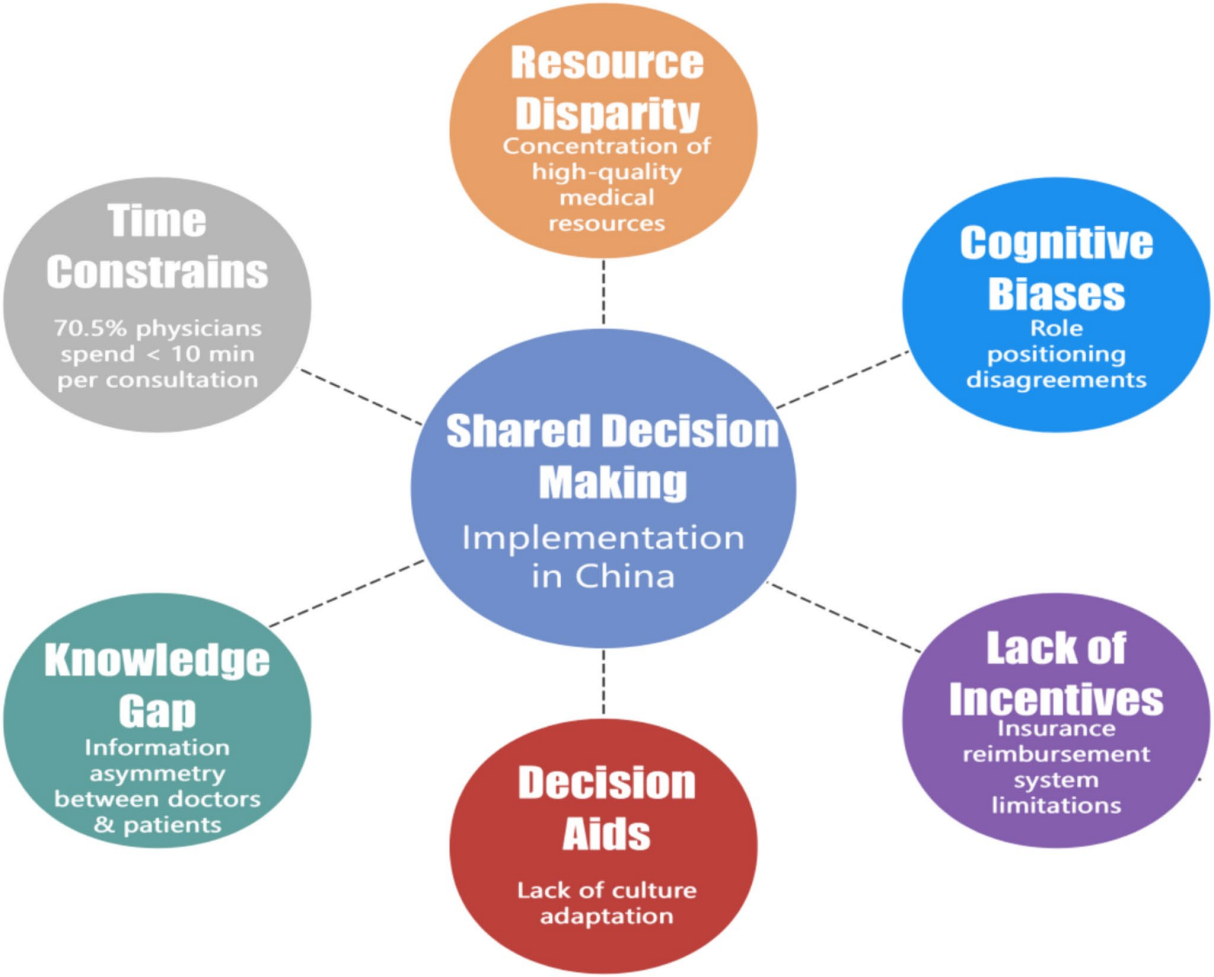
Influence factors for SDM implementation in China.

Significant regional disparities exist in China’s healthcare resource allocation, characterized by excessive concentration of high-quality resources. This maldistribution creates heavy clinical workloads. Zhang’s study revealed that 70.5% of tertiary care hospital outpatient physicians averaged ≤10 min per consultation, constraining SDM implementation (17). A critical barrier is the knowledge gap and information asymmetry between physicians and patients. Research by Li et al. indicates that patients have varying comprehension levels, and inconsistent online information complicates communication (18). Most Chinese DAs constitute direct translations without adequate cross-cultural adaptation, failing to address context-specific values and health beliefs (19). Furthermore, prevailing fee-for-service reimbursement structures and clinical productivity-focused performance metrics create insufficient incentives that disincentivize SDM adoption (20). Cognitive barriers also exist; while the philosophy of SDM has gained theoretical acceptance, substantial discrepancies persist in physician and patient perceptions of their respective roles during this process. A survey by Wang et al. found that surgical providers worried that excessive information disclosure might increase medical risks, while patients often passively accepted recommendations (21). These challenges (summarized in Figure 2) highlight the limitations of traditional SDM models and the potential for innovative technological solutions to enhance SDM practices.

### 3.3 Current applications of LLMs in SDM

In the context of multiple challenges faced in the implementation of SDM in China, technological innovation has become a key pathway for promoting the localization of SDM. The introduction of technologies such as LLMs can optimize physician-patient communication, bridge the information gap, and enhance the feasibility and practical effectiveness of SDM while respecting cultural characteristics.

In healthcare, LLMs are classified into general-purpose models (e.g., GPT series) (22) and those pre-trained for medical applications (e.g., BioBERT, ClinicalBERT, PubMedBERT) (23). Their applications include: (1) Intelligent assistant tools integrated into electronic health record (EHR) systems for information extraction and summarization (24); (2) Natural-language-based intelligent diagnostic and treatment recommendation systems, which support clinical decision-making (25); (3) Personalized patient education and decision-support platforms, enhancing patient engagement in the decision-making process (26); (4) Medical literature analysis and knowledge management tools, enabling efficient retrieval and synthesis of scientific findings (27); (5) Clinical trial design and drug-development support systems, assisting in the design and evaluation of research methodologies; and (6) Conversational agents for physician-patient communication, improving interaction efficiency and patient comprehension (28). Building on the aforementioned applications of LLMs in the healthcare field, their potential pathways for implementation in SDM can be explored.

#### 3.3.1 Information retrieval and integration

Accurate and comprehensive information exchange constitutes the foundational element of SDM. LLMs enhance this by extracting key insights from vast amounts of unstructured data, such as EHRs, clinical notes, and medical literature, providing evidence-based support for decision-making (29). By utilizing LLM technology, healthcare professionals and patients can make more informed, data-driven decisions. For example, LLMs can extract critical information like medical history, treatment records, and risk factors, automatically integrate and present these insights to both healthcare professionals and patients. This enhances information transparency and improves communication efficiency (preliminary findings) (30). Research by Du et al. indicates that LLMs streamline the extraction of clinical information from EHRs, reducing processing time and enhancing decision-making quality (preliminary findings) (31).

#### 3.3.2 Development of personalized DAs

Advancements in NLP have facilitated the development and widespread adoption of personalized patient DAs. These tools simplify medical terminology and generate patient-friendly, personalized reports, delivering tailored recommendations aligned with patient-specific needs. Amit Gupta utilized five LLMs (GPT-4o, Google Gemini, Claude Opus, Llama-3.1-8B, and Phi-3.5-mini) to simplify oncology CT reports and compared patients’ comprehension of the original versus the simplified versions (32). The results indicated that patients in the simplified report group had a significantly better understanding of the primary site and extent of their disease, and reported notably higher confidence and comprehension regarding the content of the report (32). Especially, LLM-driven interactive dialogue systems can accurately identify patient preferences, health conditions, and concerns. These systems analyze patient inputs to generate detailed explanations of treatment options, potential risks, and expected outcomes, empowering patients to actively participate in the decision-making process. For example, Brian D. Earp developed a Personalized Patient Preference Predictor (P4) based on LLMs, which has the potential to predict patients’ actual treatment preferences more accurately than current approaches that rely on surrogate decision-makers (33). These outcomes substantiate LLMs’ transformative potential in SDM and offer actionable guidance for designing culturally adaptive DAs. Studies indicate that LLMs help patients better understand their medical options, leading to more active participation in the decision-making process (34). Rietjens et al. reported that LLM-enhanced DAs dynamically adjust information delivery based on the needs of cancer patients making palliative care, increasing their perceived control and engagement in the decision-making process (35).

### 3.4 Patient sentiment analysis and psychological support

In the SDM process, patients’ emotional and psychological states significantly impact their decision-making experience and treatment adherence. LLMs use sentiment analysis to detect emotional cues like anxiety and confusion, enabling healthcare providers to adjust communication strategies and offer targeted psychological support (36). Specifically, LLM technology relies on NLP and machine learning to annotate emotional expressions in patient interactions. By analyzing linguistic cues such as tone, word choice, and sentence structure, LLMs can accurately assess a patient’s emotional state (36). For instance, if heightened anxiety is detected, the system flag this concern and notify healthcare providers. Research by Lei et al. indicates that LLM-driven sentiment analysis systems not only recognizes patient emotions but also generates tailored communication recommendations, allowing healthcare professionals to provide necessary emotional support during the decision-making process (37) and ensure patients feel understood for throughout their medical journey. Imel’s research highlights that LLM-based sentiment analysis enhances communication effectiveness in primary healthcare settings, particularly among emotionally vulnerable patients, improving their decision-making experience (38). Consequently, leveraging its sentiment analysis capabilities and personalized messaging abilities, LLMs can enhance the quality of physician-patient communication.

**Figure 3:**
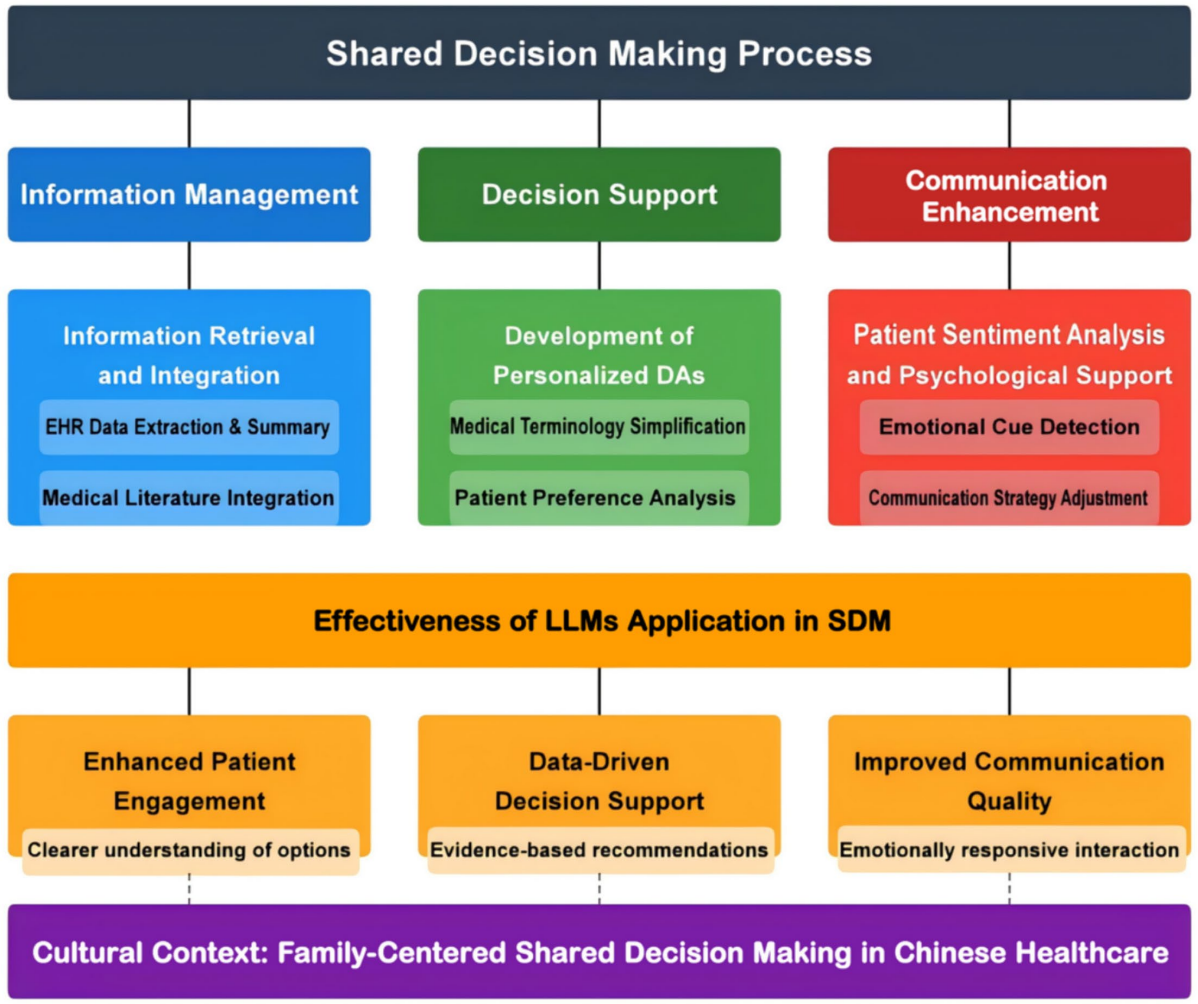
Current applications of LLMs in SDM.

In conclusion, LLMs significantly impact SDM in three areas: information extraction and integration, personalized DAs, and patient sentiment analysis. Empirical evidence demonstrates direct patient benefits in clinical settings. A systematic review synthesizing findings from seven studies revealed that for brain tumor patients, LLMs provided interactive question-answer sessions explaining the indications and side effects of surgery, radiotherapy, and chemotherapy, while adjusting information complexity based on patients’ cognitive status (15). This personalized approach yielded measurable outcomes in clinical trials, with patients using LLM-assisted decision support reporting a 23% increase in decision satisfaction and 18% improvement in treatment adherence (15). These findings directly address the need for patient-centered applications of LLM technology beyond healthcare provider assistance. LLMs boost patient engagement, provide evidence-based recommendations and improve physician-patient communication, optimizing the healthcare decision-making experience (Figure 3).

### 3.5 Challenges in implementing LLMs in SDM

#### 3.5.1 The existing universal problems

##### 3.5.1.1 The limitations of LLMs itself

Prior to the clinical deployment of large language models (LLMs), several inherent challenges must be considered. On one hand, while general-purpose LLMs leverage algorithms to produce fluent text, their deficiency in foundational medical knowledge can lead to the generation of misunderstandings and misinformation; On the other hand, general-purpose LLMs (e.g., ChatGPT) lack real-time knowledge updates (39). They are limited to retrieving information available only up to their last training date cutoff. This may lead to their mechanism becoming overloaded, resulting in a reduction in the accuracy of the information they provide. Research utilizing EHR data from 660 patients demonstrated that the accuracy of current models in managing complex clinical conditions remains to be improved (40). This suggests that researchers need to conduct more pre-training targeting specific medical and nursing domains to improve the performance of general-purpose large language models (41). Nevertheless, within the clinical medicine domain, data availability is often constrained by privacy concerns and regulatory requirements. This poses difficulties for effectively training models. Furthermore, how to avoid the biases introduced by using training data from a specific population group is also a major challenge. Data transparency is another obstacle faced by LLMs when applied to SDM. Due to the opacity regarding the sources, copyright status, and consent policies of the underlying training data, there are legal and ethical blind spots. The algorithmic “black box” of LLMs causes the transformation process from input to output to be partially or entirely hidden, resulting in a reduction in their data interpretability as well.

##### 3.5.1.2 The clinical practicality problems existing in LLMs

The practical implementation of LLMs in decision support faces practical challenges. The recommendations generated by these models often lack specific clinical guidance, requiring healthcare professionals to spend considerable time on interpretation and modification. System integration is another major challenge, as most hospital information systems are relatively closed environments, posing technical barriers to interfacing with novel AI tools. A systematic review of 26 studies by Ye et al. highlighted that the absence of standards addressing patient-generated health data (PGHD) use cases and consensus on interoperability standards results in variations in data representation and coding that impede data exchange, normalization, and integrity. Significant disparities in system compatibility markedly diminish the utility value of these tools (42). Furthermore, information exchange between different hospital departments and across healthcare institutions remains fragmented, affecting the continuity of decision support. Healthcare professionals’ acceptance of LLM-based systems also presents a significant barrier. Despite ongoing technological advancements, studies indicate that over 60% of healthcare providers are hesitant to adopt AI-driven systems due to concerns about transparency and data security. Although Explainable AI (XAI) has improved the interpretability of these models, lingering concerns regarding model transparency and data security continue to hinder adoption (43). This skepticism directly impacts the effectiveness of AI-driven decision support in clinical practice.

#### 3.5.2 The adaptability issues of LLMs in China

The application of LLMs in China’s healthcare system faces significant localization challenges. One major issue is linguistic and cultural adaptation. Most LLMs are trained on English-language corpora, leading to difficulties in processing Chinese medical terminology, particularly in traditional Chinese medicine (TCM). Research by Cao et al. found that non-localized models had an accuracy rate below 60% in TCM diagnostic dialogues, often misinterpreting symptom and syndrome differentiation (44).

Another challenge is the adaptation of decision-making model. Influenced by Confucian culture, the family plays a central role in medical decisions (19), often limiting patient autonomy, especially in cases of major illnesses and geriatric care (45). This family-centered decision-making model conflicts with the individual autonomy emphasized by modern medicine, necessitating that SDM respects patient preferences while also considering family involvement. In China, medical decision-making typically involves multiple stakeholders, including family members and social networks, complicating the decision process and increasing demands on LLMs.

Additionally, regional adaptability is crucial in China due to uneven healthcare resource distribution and varying medical conditions across regions. The effectiveness of LLMs in China may align with findings from a multi-center study by Gulnoza et al., which emphasized the need for AI decision-support systems to adapt to socio-cultural and dietary differences, particularly in Central Asia (46). This underscores the importance of considering regional disparities in healthcare infrastructure for more flexible AI decision-making.

Lastly, compatibility with China’s healthcare insurance policies poses another challenge. China’s complex medical insurance and reimbursement system influences clinical decision-making. Research by He et al. highlighted that current AI decision support systems often overlook key policy factors such as reimbursement restrictions and the hierarchical medical system, leading to discrepancies between AI recommendations and actual clinical practices (47). To enhance practical utility, future optimizations should integrate healthcare policy constraints and align recommendations with China’s medical system.

## 4 Discussion and future directions

Considering the characteristics of Chinese healthcare system, the optimization of LLMs should focus on integrating domain-specific knowledge tailored to the Chinese medical landscape.

### 4.1 Integration of traditional Chinese medicine and biomedicine knowledge

Most current LLMs are mainly trained on Western medical knowledge, limiting their understanding of TCM theories and practices. To improve their applicability, a combined knowledge graph of biomedicine and TCM is recommended to enhance interpretation of TCM terminology, syndrome differentiation, and treatment methodologies. Research by Ren Haiyan et al. on the standardization of TCM terminology suggests that integrating a TCM ontology-based knowledge base with deep learning techniques can significantly enhance model accuracy in interpreting TCM clinical dialogues (48). Additionally, enhancing the model’s understanding of culturally specific symptom descriptions in Chinese contexts would improve the accuracy of medical history collection.

### 4.2 Optimization of multilevel expression capabilities

Due to health literacy disparities among Chinese patients, LLMs should adjust their language complexity to individual cognitive levels. Zhou et al.’s multilevel medical knowledge framework indicates that a progressive explanation strategy enhances comprehension across different education levels (49). Furthermore, improving dialect comprehension is vital in primary healthcare, as it can increase patient engagement in rural areas.

### 4.3 Enhancing decision-making transparency and traceability

Improving the integrity of the decision-making process is another critical area for LLMs optimization. Besides generating decision recommendations, these models should offer explanations to justify their suggestions, enabling providers to audit the rationale and ensure compliance with clinical guidelines. A study on the interpretability of neural network models by Han Wei et al. found that systems with decision traceability capabilities significantly outperformed black-box models in clinical usability (50).

### 4.4 Optimization of family-inclusive decision-making support

Given China’s family-centered healthcare decision-making model, AI-driven SDM tools should support enable collaborative platforms for remote family participation and provide a comprehensive record of the decision-making process. Such platforms would better accommodate the socio-cultural context of Chinese healthcare and improve patient and family engagement in shared medical decisions.

### 4.5 The role of LLMs in China’s healthcare reform

As China’s healthcare reform deepens, LLM-driven SDM is expected to promote the fairness, efficiency, and quality of healthcare services. China’s healthcare reform emphasizes goals such as hierarchical diagnosis and treatment, improving the capabilities of primary healthcare services, and promoting “Internet+Healthcare.” The application of LLMs in these areas can significantly enhance the capacity of primary healthcare services and support the implementation of hierarchical diagnosis and treatment. For example, LLMs, by incorporating evidence-based medical evidence, can help bridge the gap in primary healthcare experience and assist patients in understanding treatment options (51). Additionally, through integration with remote consultation systems, LLMs can facilitate efficient communication between primary healthcare providers and specialists from higher-level hospitals, optimizing referral processes. Furthermore, LLMs can optimize medical resource allocation and reduce costs. By analyzing patient symptoms, LLM-driven dialogue systems can assist with consultations, medical history collection (52), diagnostic recommendations, and more, alleviating the workload of healthcare providers and minimizing the waste of medical resources. By providing transparency in the rationale behind treatment decisions (53), LLMs help both patients and healthcare providers collaboratively choose more cost-effective treatment plans, thereby reducing the burden on health insurance.

## 5 Conclusion

This study systematically analyzed the application, challenges, and future prospects of LLMs in SDM within China’s healthcare system. The findings indicate that SDM in China faces unique challenges, including cultural traditions, family-centered decision-making models, and knowledge gap, while also constrained by healthcare resource disparities and limited primary care capacity. As an emerging technological tool, LLMs demonstrated significant potential in improving decision-making efficiency, optimizing information transmission, and enhancing patient engagement, particularly in addressing the supply–demand imbalance in China’s healthcare system. However, the application of LLMs in SDM remains in an exploratory stage. From a technical perspective, further advancements are needed in integrating TCM knowledge, enhancing multi-level expression capabilities, improving the interpretability of decision-making processes and optimizating of family-inclusive decision-making support. As China’s healthcare reform continues to advance, the application of LLMs will further expand. Through ongoing optimization of algorithms and datasets, LLMs will play an increasingly significant role across all stages of healthcare service delivery. Looking ahead, as technology matures and practical experience accumulates, LLMs have the potential to become a crucial driving force in advancing SDM in China, ultimately contributing to a more patient-centered, efficient, and culturally adaptive healthcare decision-making process.
